# A Refined Theory for Characterizing Adhesion of Elastic Coatings on Rigid Substrates Based on Pressurized Blister Test Methods: Closed-Form Solution and Energy Release Rate

**DOI:** 10.3390/polym12081788

**Published:** 2020-08-10

**Authors:** Yong-Sheng Lian, Jun-Yi Sun, Zhi-Hang Zhao, Shou-Zhen Li, Zhou-Lian Zheng

**Affiliations:** 1 School of Civil Engineering, Chongqing University, Chongqing 400045, China; lianyongsheng@cqu.edu.cn (Y.-S.L.); 20135542@cqu.edu.cn (Z.-H.Z.); 201816021070@cqu.edu.cn (S.-Z.L.); zhengzl@cqu.edu.cn (Z.-L.Z.); 2 Key Laboratory of New Technology for Construction of Cities in Mountain Area (Chongqing University), Ministry of Education, Chongqing 400045, China

**Keywords:** film/substrate delamination, pressurized blister test, circular membrane, closed-form solution, energy release rate

## Abstract

Adhesion between coatings and substrates is an important parameter determining the integrity and reliability of film/substrate systems. In this paper, a new and more refined theory for characterizing adhesion between elastic coatings and rigid substrates is developed based on a previously proposed pressurized blister method. A compressed air driven by liquid potential energy is applied to the suspended circular coating film through a circular hole in the substrate, forcing the suspended film to bulge, and then to debond slowly from the edge of the hole as the air pressure intensifies, and finally to form a blister with a certain circular delamination area. The problem from the initially flat coating to the stable blistering film under a prescribed pressure is simplified as a problem of axisymmetric deformation of peripherally fixed and transversely uniformly loaded circular membranes. The adhesion strength depends on the delamination area and is quantified in terms of the energy released on per unit delamination area, the so-called energy release rate. In the present work, the problem of axisymmetric deformation is reformulated with out-of-plane and in-plane equilibrium equations and geometric equations, simultaneously improved, and a new closed-form solution is presented, resulting in the new and more refined adhesion characterization theory.

## 1. Introduction

Coating technology is indispensable in many applications fields [1,2,3,4,5]. A film/substrate system usually consists of a substrate and its thin coating film, where the substrate could be a rigid substrate or a soft substrate, while the coating film is usually formed by chemical synthesis methods or physical deposition techniques and is usually polymer thin films, also including some polymer-based composite coatings with specific properties such as high adherence, crystallinity, temperature and corrosion resistance, or high conductivity [6,7]. The integrity and reliability of film/substrate systems obviously depend on the adhesion strength of coatings on substrates, which is often quantified or defined in terms of the energy released on per unit film/substrate delamination area, i.e., the so-called energy release rate. Various methods, such as blister tests [8,9,10], peeling tests [11,12], and the centrifugal adhesion test [13,14], have been developed to realize the delamination of coating films from substrates. Blister tests and peeling tests are two methods often used for the delamination between thin elastic coating films and rigid substrates. However, compared with the peeling method, the blister test technique has the advantages of axisymmetric blister geometry and small angle at the crack front [15].

**Figure 1:**
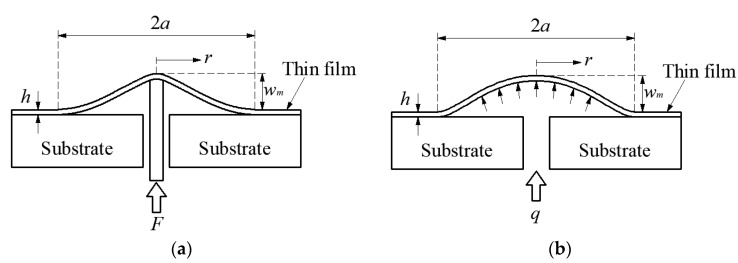
Sketch of the loading configurations for (**a**) shaft-loaded blister, (**b**) pressurized blister [9].

Dannenberg initially proposed using the blister test technique to characterize the interface adhesion strength [15]. In a blister test, a hole is bored or chemically etched in the rigid substrate until it reaches the thin film, and then a crack driving force is applied gradually through the hole until an axisymmetric blister crack extends into the interface of the thin film/substrate system. Then, the energy release rate can be determined by measuring the crack driving force, the radius and height of the blister. The blister test was developed into many different forms by subsequent researchers [16,17,18,19,20], in which two kinds of crack driving force are usually adopted, that is the shaft-loading as shown in Figure 1a and the gas or fluid pressure loading as shown in Figure 1b. In the shaft-loaded blister test, the radius of the loading-shaft is usually very small, which may lead to plastic yielding and piercing of the thin film. In the pressurized blister test, the catastrophic debonding will occur once the applied load exceeds the critical pressure [15], so a precise experimental setup is needed to simultaneously monitor the size of the blister and the applied pressure.

**Figure 2:**
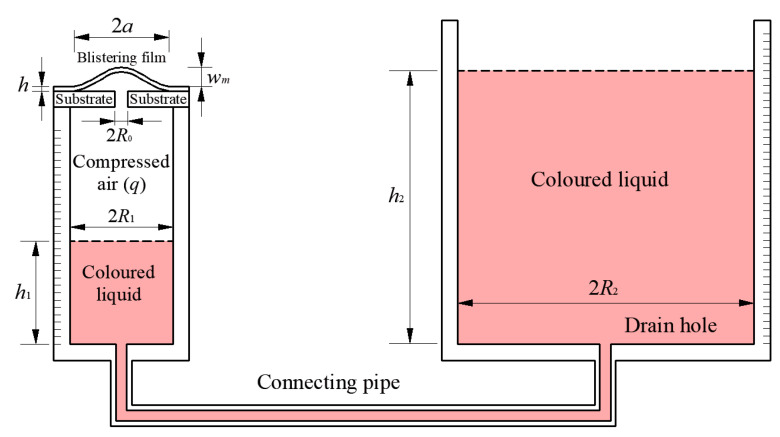
Schematic view of the experimental setup for pressurized blister test [9].

In previous work, we developed a novel loading method for pressurized blister test and presented the formula of the energy release rate [9], the experimental setup used is shown in Figure 2, where *h*, *a* and *w_m_* are the thickness, radius and maximum deflection of the blistering film, *R*_0_ is the radius of the hole in the rigid substrate, *R*_1_ and *R*_2_ are the inner radii of the smaller and lager circular containers, *h*_1_ and *h*_2_ are the height of liquid in the two circular containers. In Sun et al. [9], the problem of axisymmetric deformation of the pressurized blistering film was simplified into the problem of axisymmetric deformation of a peripherally fixed and transversely uniformly loaded circular membrane, i.e., the well-known Hencky problem. The well-known Hencky solution was used to derive the formula of the strain energy stored in the blistering film [21]. Some assumptions or approximations were adopted in the derivation of the well-known Hencky solution: (i) the so-called small-rotation-angle assumption of membrane was used to derive the so-called out-of-plane equilibrium equation; (ii) the effect of the deflection on the so-called in-plane equilibrium was ignored during the derivation of the in-plane equilibrium equation; (iii) the geometric equation is established by assuming that before and after the deformation of the membrane the length of the micro line element is approximately equal and omitting the term ${\left(du/dr\right)}^{2}$. These assumptions or approximations could make it convenient to obtain the analytical solution of the well-known Hencky problem, but they will also make the well-known Hencky solution not work when the deflection is relatively large. In a pressurized blister test, however, the deflection of the blistering thin film could reach half the radius of the circular blistering thin film, or even larger, so it is necessary to give up these assumptions or approximations when accurately analyzing the elastic response of the pressurized blistering thin film. Moreover, there are some errors in the derivation of the energy release rate in Sun et al. [9]. For example, the integral mean value theorem for calculating mean value of a curve should not be used to calculate the volume under the blistering thin film when determining the strain energy stored in the blistering thin film, and the method to determine the work caused by the change of liquid potential energy also needs to be improved.

In this paper, an overall improvement in experiment and theory for characterizing the adhesion between rigid substrates and elastic coatings in Sun et al. [9] is made. In the following section, some improvements in the experimental setup shown in Figure 2 are made, and the measurement process is described in detail. In Section 3, some errors in the derivation of the formula of the energy release rate in Sun et al. [9] are rectified, and in order to obtain a more accurate formula of the energy release rate, the external work caused by the change of the potential energy of the colored liquid in the two containers and the energy absorbed by the enclosed air due to being compressed are accurately derived, and based on the obtained new and more refined closed-form solution for the problem of axisymmetric deformation of a peripherally fixed and transversely uniformly loaded circular membrane the elastic strain energy stored in the blistering thin film is also derived, and finally a more accurate formula of the energy release rate is presented; In Section 4, the validity of the obtained new refined closed-form solution is demonstrated by a comparison with the results obtained by the conducted experiment, and the influence of the above-mentioned assumptions or approximations (i)–(iii) on the closed-form solution of the well-known Hencky problem is investigated, also the difference between the calculation results of the strain energy stored in the blistering film obtained by the well-known Hencky solution and by the new refined closed-form solution presented here was discussed. Concluding remarks are presented in Section 5.

## 2. Methods

**Figure 3:**
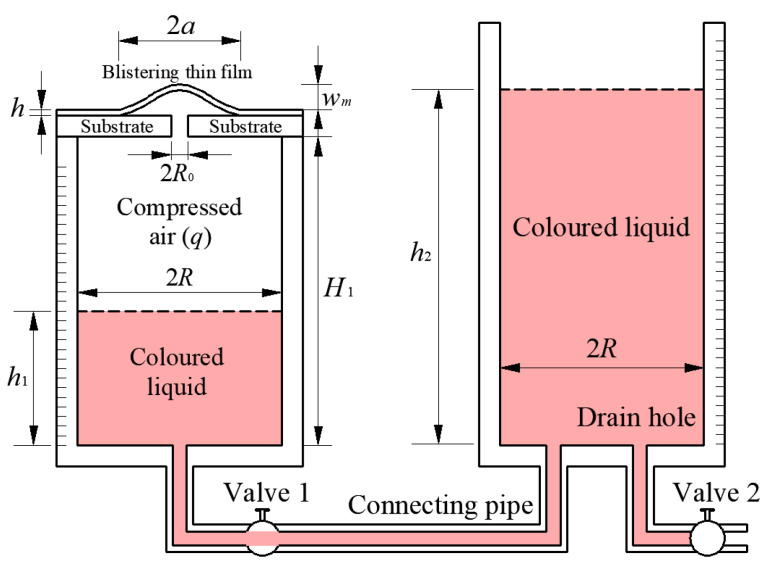
Schematic view of the improved experimental setup for pressurized blister test.

The experimental setup shown in Figure 2 still needs to make some improvement. In this experimental setup, the circular containers are designed with different inner radii, so that more pressure can be applied to the thin film. However, as it is known, in one and the same liquid, the same pressure is present at the same height. It is also valid for the pressure at the air and liquid interface. So, the relative pressure (relative to ambient pressure) of the compressed air *q* is only dependent on the density of the liquid *ρ*, the acceleration of gravity g, and the height difference of liquid in the two containers $h_{1}-h_{2}$, and independent of the inner radius of the circular container [22]. So, it’s not necessary to design the two circular containers with different inner radii, and different inner radii will make the following derivation process of the energy release rate complicated. In addition, it is necessary that a valve is added to the connecting pipe, such that the influence of the fluctuation of liquid caused by pouring liquid into the right container during the experiment can be avoided by closing the valve. The experimental setup after being improved is shown in Figure 3, where *R* is the inner radius of the two circular containers.

**Figure 4:**
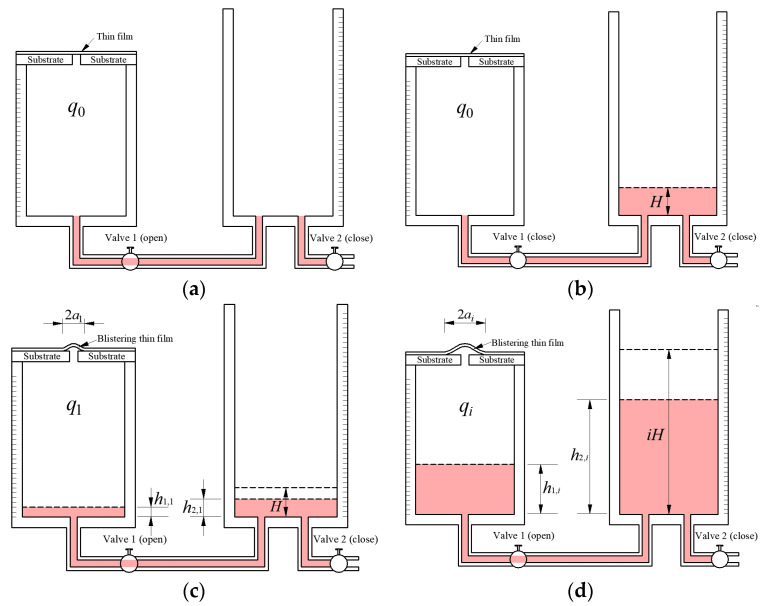
Schematic view of loading process or method of pressurized blister test: (**a**) Initial state; (**b**) Status before opening the valve 1 in step 1 of loading operation; (**c**) Stable state after step 1 of loading operation; (**d**) Stable state after step *i* of loading operation.

For the improved experimental setup shown in Figure 3, the experimental operating steps and measurement method of a pressurized blister test is detailed as follows:

1. Open valve 1 and close valve 2, and fill the connecting and drainpipes with colored liquid. Then, a specimen of a thin film/substrate system is tightly adhered to the left container, as shown in Figure 4a. At this time, the pressure $q_{0}$ of the enclosed air in the left container is assumed to be equal to zero.
2. Close valve 1 and pour colored liquid with volume $\pi R^{2}H$ into the right container, as shown in Figure 4b. Since valve 1 is closed, the pressure of the enclosed air in the left container remains $q_{0}$.
3. Open valve 1, and the colored liquid in the right container will slowly flow into the left one. After the height of colored liquid is stabilized, the height of colored liquid in the left container becomes $h_{1,1}$, and the height of colored liquid in the right container becomes $h_{2,1}=H-h_{1,1}$, as shown in Figure 4c. The relative pressure of the enclosed compressed air in the left container relative to ambient pressure becomes $q_{1}$
  (1) $$q_{1}=\rho g(h_{2,1}-h_{1,1})=\rho g(H-2h_{1,1}).$$
  In addition, under the action of pressure $q_{1}$, a blistering thin film with radius of $a_{1}$ appears. This is the first loading, i.e., the step 1 of loading operation.
4. Repeat the operations in 2 and 3, i.e., close valve 1 again and pour colored liquid with volume $\pi R^{2}H$ into the right container and then open valve 1. After step *i* of loading operation, the colored liquid with total volume $i\pi R^{2}H$ (*i* = 2, 3, 4, …, *n*) is poured into the right container, the height of liquid in the left container becomes $h_{1,i}$, and the height of liquid in the right container becomes $h_{2,i}$, and
  (2) $$h_{2,i}=iH-h_{1,i}.$$
  The relative pressure of the compressed air in the left container becomes $q_{i}$
  (3) $$q_{i}=\rho g(h_{2,i}-h_{1,i})=\rho g(iH-2h_{1,i}).$$
  Under the action of pressure $q_{i}$, the radius of the blistering thin film becomes $a_{i}$, as shown in Figure 4d.
5. After step *n* of loading operation, the radius of the blistering thin film is assumed to become *a*. Record the height of the liquid in the two containers and the radius of the blistering thin film after each step of loading operation, and open valve 2 to drain all the liquid in the right container to see if the blistering thin film fully becomes flat. If the blistering thin film is quick and fully becomes flat, then this indicates that the blistering thin film works within the elastic range, otherwise the results of this test are invalid.

## 3. Energy Release Rate

In the pressurized blister test shown in Figure 3 and Figure 4, based on the law of conversation of energy the external work caused by the change of the potential energy of the colored liquid in the two containers (which is denoted by *U_F_*) may be assumed to be completely converted into the energy absorbed by the enclosed air due to being compressed (which is denoted by *U_a_*), the elastic strain energy stored in the blistering thin film (which is denoted by *U_ef_*), and the energy released on the delamination region (which is denoted by *U_d_*), that is *U_F_* =*U_a_* +*U_ef_* +*U_d_*. Suppose that the area of the delamination region is denoted as *S*. Obviously, *S* is a function of the radius *r* of the circular delamination region, that is $S=\pi (r^{2}-R_{0}^{2})$ (in which $R_{0}\leq r\leq a$), hence $0\leq S(r)\leq \pi (a^{2}-R_{0}^{2})$. The energy released on the delamination region, *U_d_*, depends on the size of the delamination region, in other words, *U_d_* is a function of the area of the delamination region *S* and may be denoted as *U_d_*(*S*). It is not difficult to understand that for the case of delamination of the coating thin film adhered uniformly to a rigid substrate, *U_d_*(*S*) can be expressed as a linear function of the area of the delamination region area *S*, that is, *U_d_*(*S*) = *KS*, in which *K* is a proportional coefficient. The adhesion strength can be quantified in terms of the energy released on per unit delamination area [9], that is, the so-called energy release rate (denoted by *G*). Therefore, the energy release rate for the case of delamination of the coating thin film adhered uniformly to a rigid substrate can be written as

(4) $$G=\frac{dU_{d}(S)}{dS}=K.$$

Note that *U_d_* = *U_F −_ U_a −_ U_ef_*, and when *r* = *a*, $S(a)=\pi (a^{2}-R_{0}^{2})$ and *U_d_*(*S*(*a*)) = *U_F_*(*a*) _−_ *U_a_*(*a*) _−_ *U_ef_*(*a*), in which *U_F_*(*a*), *U_a_*(*a*) and *U_ef_*(*a*) denote their values corresponding to *r* = *a*. Hence, from *U_d_*(*S*) = *KS* it is found that

(5) $$K=\frac{U_{d}(S(a))}{S(a)}=\frac{U_{F}(a)-U_{a}(a)-U_{ef}(a)}{\pi (a^{2}-R_{0}^{2})}.$$

Therefore, the energy release rate here can finally be written as

(6) $$G=\frac{U_{F}(a)-U_{a}(a)-U_{ef}(a)}{\pi (a^{2}-R_{0}^{2})}.$$

In this way, based on the experimental setup shown in Figure 3 and Figure 4, the energy release rate here can be determined with the measured values of the maximum radius *a* of the circular delamination region and the height *h_1_*_,*i*_ of the liquid in the left container after each step of loading operation.

However, the formula of the energy release rate presented in Sun et al. [9] is not accurate enough and also not applicable when the deflection of the blistering thin film is relatively large, because the well-known Hencky solution used in the derivation of the formula of the energy release rate is obtained based on the above-mentioned assumptions or approximations (i)–(iii). In addition, there are some errors in the derivation of the formula of the energy release rate in Sun et al. [9]. These errors will be rectified in the following, and in order to obtain a more accurate formula of the energy release rate, the external work *U_F_*(*a*), the energy *U_a_*(*a*), and the elastic strain energy *U_ef_*(*a*) are respectively derived as follow.

### 3.1. The External Work U_F_(a)

Since the liquid is poured into the right container by many times and the height of liquid in the right container changes after the valve 1 is opened, the external work caused by the change of liquid potential energy in each step of loading operation should be calculated separately, rather than the calculation method in Sun et al. [9]. In the stable state after step *i*−1 of loading operation, the height of liquid in the two containers are $h_{1,i-1}$ and $h_{2,i-1}$ respectively. While in step *i* of the loading operation, the colored liquid with volume $\pi R^{2}H$ is poured into the right container, and the height of liquid in the two containers are $h_{1,i}$ and $h_{2,i}$ after opening the valve 1. So, if the plane at the bottom of the container is taken as the zero potential energy plane, the external work *U_F,i_* caused by the change of liquid potential energy in the step *i* of loading operation can be written as

(7) $$U_{F,i}=\frac{1}{2}\pi \rho gR^{2}h_{1,i-1}^{2}+\frac{1}{2}\pi \rho gR^{2}{\left(h_{2,i-1}+H\right)}^{2}-\frac{1}{2}\pi \rho gR^{2}h_{1,i}^{2}-\frac{1}{2}\pi \rho gR^{2}h_{2,i}^{2},$$

where *ρ* denotes the density of the liquid in the container, *g* denotes the acceleration of gravity, *R* denotes the inner radius of the two circular containers, and *H* denotes the height of the liquid added to the right container in each step of loading operation. By means of Equation (2), Equation (7) gives

(8) $$\begin{array}{ll} U_{F,i} & =\frac{1}{2}\pi \rho gR^{2}h_{1,i-1}^{2}+\frac{1}{2}\pi \rho gR^{2}{\left[(i-1)H-h_{1,i-1}+H\right]}^{2}-\frac{1}{2}\pi \rho gR^{2}h_{1,i}^{2}-\frac{1}{2}\pi \rho gR^{2}{\left(iH-h_{1,i}\right)}^{2}\\ & =\pi \rho gR^{2}[h_{1,i-1}^{2}-h_{1,i}^{2}-iH(h_{1,i-1}-h_{1,i})] \end{array}$$

Suppose that the radius of the blister reaches *a* after *n* times loading operations, then the *U_F_*(*a*) can be written as

(9) $$\begin{array}{ll} U_{F}(a) & =\sum_{i=1}^{n}U_{F,i}=\pi \rho gR^{2}\sum_{i=1}^{n}\left[h_{1,i-1}^{2}-h_{1,i}^{2}-iH(h_{1,i-1}-h_{1,i})\right]\\ & =\pi \rho gR^{2}(H\sum_{i=1}^{n-1}h_{1,i}-nHh_{1,n}-h_{1,n}^{2}) \end{array}$$

### 3.2. The Energy U_a_(a)

Since the loading operation is very slow in each step, the compression of the enclosed air in the left container can be regarded as isothermal compression, where the internal energy of the enclosed air remains unchanged. So, the energy *U_a_*(*a*) absorbed when the air is compressed will be released into the atmosphere in the form of heat energy. In the process of isothermal compression of the air in left container, the temperature *T* of the air remains constant. So,

(10) PV=NkT=constant,

where *P* is the pressure of gas, *V* is the volume of gas, *N* is the number of gas molecules per unit volume, and *k* is Boltzmann constant. Then, *U_a_*(*a*) can be written as

(11) $$\begin{array}{ll} U_{a}(a) & =-\int_{V_{0}}^{V_{n}}PdV=-NkT\int_{V_{0}}^{V_{n}}\frac{1}{V}dV=NkT\ln\frac{V_{0}}{V_{n}}\\ & =NkT\ln\frac{H_{1}}{H_{1}-h_{1,n}} \end{array}$$

where $V_{0}$ ($V_{0}=\pi R^{2}H_{1}$) and $H_{1}$ are the initial volume and height of the air in left container, $V_{n}$ ($V_{n}=\pi R^{2}H_{1}-\pi R^{2}h_{1,n}$) and $h_{1,n}$ are the volume of the air and the height of the colored liquid in left container after the loading operation of step *n*.

### 3.3. The Elastic Strain Energy U_ef_(a)

**Figure 5:**
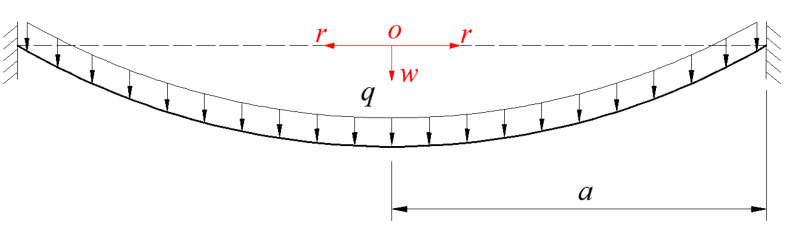
Sketch of the circular membrane under transverse loads *q*.

When determining the elastic strain energy *U_ef_*(*a*) stored in the blistering thin film, the exact analytical solution for the problem of axisymmetric deformation of the pressurized blistering thin film should be obtained first. This problem can be regarded as the well-known Hencky problem, as shown in Figure 5, whereby a transverse uniformly distributed loads *q* is applied onto the surface of a linearly elastic, initially flat, peripherally fixed circular membrane with radius *a*. The analytical solution used in Sun et al. [9] is exactly the well-known Hencky solution, where the above-mentioned assumptions (i)–(iii) are adopted. By giving up the so-called small-rotation-angle assumption (i.e., the assumption (i)), Sun et al. re-solved the well-known Hencky problem and obtained its power series solution [23], and solved the problem of axisymmetric deformation of prestressed Föppl-Hencky membrane under constrained deflecting [24]. Yang et al. [15] obtained the closed-form solution of the axisymmetric deformation problem of prestressed membrane also by giving up the so-called small-rotation-angle assumption. Recently, Sun et al. [25] presented a new closed-form solution of the well-known Hencky problem by simultaneously giving up the assumptions (i) and (ii), but the assumption (iii) is still where it is. So, it is necessary to take in account the effect of the deflection on the in-plane equilibrium equation, and give up the approximations used in the geometric equation.

**Figure 6:**
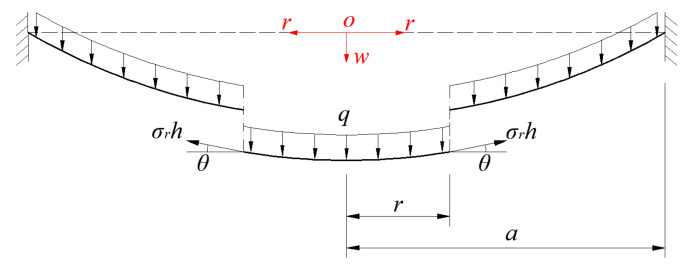
Sketch of the static equilibrium of the isolated-body.

Now, let us take a rotationally symmetric isolated-body with radius *r* (0<r≤a) from the central portion of the deformed membrane, with a view of studying its static equilibrium problem under the joint action of loads *q* and the membrane force $\sigma_{r}h$ acted on its boundary, as shown in Figure 6, where *h* is thickness, $\sigma_{r}$ is radial stress, θ is the slope angle of the deflected membrane.

In the vertical direction perpendicular to the initially flat circular membrane, there are two vertical forces, i.e., the total force $\pi r^{2}q$ (0<r≤a) of the uniformly distributed loads *q* within radius *r*, and the total vertical force $2\pi r\sigma_{r}h\sin\theta$ produced by the vertical component of the radial membrane force $\sigma_{r}h$. So, the out-of-plane equilibrium equation can be written as,

(12) $$2\pi r\sigma_{r}h\sin\theta =\pi r^{2}q,$$

where,

(13) $$\sin\theta =1/\sqrt{1+1/\tan^{2}\theta }=1/\sqrt{1+1/{\left(-\frac{dw}{dr}\right)}^{2}}.$$

Substituting Equation (13) into Equation (12), it is found that

(14) $$\frac{1}{2}rq\sqrt{1+1/{\left(-\frac{dw}{dr}\right)}^{2}}=\sigma_{r}h.$$

By taking in account the effect of the deflection on the in-plane equilibrium equation, a new in-plane equilibrium equation is obtained, that is

(15) $$\frac{d}{dr}(r\sigma_{r})-\sigma_{t}[1+{\left(\frac{dw}{dr}\right)}^{2}]=0,$$

where the term $\left[1+{\left(dw/dr\right)}^{2}\right]$ represents the effect of deflection on the in-plane equilibrium equation and $\sigma_{t}$ is the circumferential stress. The detailed derivation of Equation (15) is shown in Appendix A. Moreover, if the approximations adopted in the geometric equations are further given up, the geometric equations may be written as [25]

(16) $$e_{r}={\left[{\left(1+\frac{du}{dr}\right)}^{2}+{\left(\frac{dw}{dr}\right)}^{2}\right]}^{1/2}-1$$

and

(17) $$e_{t}=\frac{u}{r},$$

where $e_{r}$ is the radial strain, $e_{t}$ is the circumferential strain, and *u* is the radial displacement. Further, if the change in membrane thickness is ignored, then the relations of the stress and strain may be written as,

(18) $$\sigma_{r}=\frac{E}{1-\nu^{2}}(e_{r}+\nu e_{t})$$

and

(19) $$\sigma_{t}=\frac{E}{1-\nu^{2}}(e_{t}+\nu e_{r}),$$

where *E* is the Young’s modulus of elasticity and ν is the Poisson’s ratio. If substituting Equations (16) and (17) into Equations (18) and (19), then it is found that

(20) $$\sigma_{r}=\frac{E}{1-\nu^{2}}[\sqrt{{\left(1+\frac{du}{dr}\right)}^{2}+{\left(\frac{dw}{dr}\right)}^{2}}-1+\nu \frac{u}{r}]$$

and

(21) $$\sigma_{t}=\frac{E}{1-\nu^{2}}[\frac{u}{r}+\nu \sqrt{{\left(1+\frac{du}{dr}\right)}^{2}+{\left(\frac{dw}{dr}\right)}^{2}}-\nu ].$$

By means of Equations (20) and (21), it is found that

(22) $$\frac{u}{r}=\frac{1}{Eh}(\sigma_{t}h-\nu \sigma_{r}h).$$

Substituting the *u* of Equation (22) into Equation (20) yields

(23) $${\left(\frac{1}{E}\sigma_{r}-\frac{\nu }{E}\sigma_{t}+1\right)}^{2}-{\left[\frac{1}{E}\frac{d}{dr}(r\sigma_{t})-\frac{\nu }{E}\frac{d}{dr}(r\sigma_{r})+1\right]}^{2}-{\left(\frac{dw}{dr}\right)}^{2}=0.$$

The boundary conditions are

(24) $$\frac{u}{r}=\frac{1}{Eh}(\sigma_{t}h-\nu \sigma_{r}h)=0\;\mathrm{at}\;r=a$$

and

(25) w=0 at r=a.

Let us introduce the following dimensionless variables

(26) $$Q=\frac{aq}{Eh},\;W=\frac{w}{a},\;U=\frac{u}{a},\;S_{r}=\frac{\sigma_{r}}{E},\;S_{t}=\frac{\sigma_{t}}{E},\;x=\frac{r}{a}.$$

By using Equation (26), Equations (14), (15) and (23) can be transformed into

(27) $$(4S_{r}^{2}-x^{2}Q^{2}){\left(\frac{dW}{dx}\right)}^{2}-x^{2}Q^{2}=0,$$

(28) $$\frac{d}{dx}(xS_{r})-S_{t}[1+{\left(\frac{dW}{dx}\right)}^{2}]=0$$

and

(29) $${\left(S_{r}-\nu S_{t}+1\right)}^{2}-{\left[\frac{d}{dx}(xS_{t})-\nu \frac{d}{dx}(xS_{r})+1\right]}^{2}-{\left(\frac{dW}{dx}\right)}^{2}=0,$$

and the boundary conditions, i.e., Equations (24) and (25), can be transformed into

(30) $$\frac{U}{x}=S_{t}-\nu S_{r}=0\mathrm{at}x=1$$

and

(31) $$W=0\mathrm{at}x=1.$$

Expand *W*, $S_{r}$, and $S_{t}$ into the power series of the *x*, i.e., let

(32) $$S_{r}=\sum_{i=0}^{\infty }b_{i}x^{i},$$

(33) $$S_{t}=\sum_{i=0}^{\infty }c_{i}x^{i}$$

and

(34) $$W=\sum_{i=0}^{\infty }d_{i}x^{i}.$$

After substituting Equations (32)–(34) into Equations (27)–(29), it can be found that, $b_{i}\equiv 0$ (i=1,3,5,⋯), $c_{i}\equiv 0$ (i=1,3,5,⋯) and $d_{i}\equiv 0$ (i=1,3,5,⋯), and the coefficients $b_{i}$ (i=2,4,6,⋯), $c_{i}$ (i=0,2,4,⋯) and $d_{i}$ (i=2,4,6,⋯) can be expressed into the polynomial with regard to the coefficient $b_{0}$, which can be found in Appendix B.

The coefficients $b_{0}$ and $d_{0}$, as the undetermined constants, can be determined by using the boundary conditions Equations (30) and (31). From Equations (32) and (33), Equation (30) gives

(35) $$\sum_{i=0}^{\infty }c_{i}-\nu \sum_{i=0}^{\infty }b_{i}=0.$$

From Equation (34), Equation (31) gives

(36) $$d_{0}=-\sum_{i=1}^{\infty }d_{i}.$$

After substituting the expressions of $b_{i}$ and $c_{i}$ into Equation (35), we can obtain an equation containing only $b_{0}$, then the undetermined constant $b_{0}$ can be determined. With this known $b_{0}$, the other undetermined constant $d_{0}$ can be determined by substituting the expressions of $d_{i}$ into Equation (36). Further, with the known $b_{0}$ and $d_{0}$, all the coefficients $b_{i}$, $c_{i}$, and $d_{i}$ can easily be determined, and the expression of $S_{r}$, $S_{t}$, and *W* can thus be presented.

From Equations (26) and (36), Equation (34) can be written as

(37) $$w(r)=a\sum_{i=1}^{\infty }d_{i}[{\left(\frac{r}{a}\right)}^{i}-1].$$

Then, the volume under the blistering thin film can be written as

(38) $$V=\int_{0}^{a}2\pi rw(r)dr=2\pi a^{2}\int_{0}^{a}\sum_{i=1}^{\infty }d_{i}[{\left(\frac{r}{a}\right)}^{i+1}-\frac{r}{a}]dr=2\pi a^{3}\sum_{i=1}^{\infty }d_{i}(\frac{1}{i+2}-\frac{1}{2}).$$

After *n* times loading operations were performed, the pressure *q* applied on the blistering thin film is

(39) $$q=\rho g(h_{2,n}-h_{1,n})=\rho g(nH-2h_{1,n}).$$

Based on the research findings [26,27], the elastic strain energy stored in the blistering thin film with the volume *V* under the loads *q* should be equal to *Vq*/4. Hence, Equations (38) and (39) give

(40) $$U_{ef}(a)=\frac{1}{4}Vq=\frac{1}{2}\pi \rho ga^{3}(nH-2h_{1,n})\sum_{i=1}^{\infty }d_{i}(\frac{1}{i+2}-\frac{1}{2}).$$

### 3.4. The Accurate Formula of Energy Release Rate

Substituting Equations (9), (11) and (40) into Equation (6), it can be obtained that

(41) $$\begin{array}{ll} G & =\frac{\rho gR^{2}}{a^{2}-R_{0}^{2}}(H\sum_{i=1}^{n-1}h_{1,i}-nHh_{1,n}-h_{1,n}^{2})-\frac{NkT}{\pi (a^{2}-R_{0}^{2})}\ln\frac{H_{1}}{H_{1}-h_{1,n}}\\ & -\frac{\rho ga^{3}}{2(a^{2}-R_{0}^{2})}(nH-2h_{1,n})\sum_{i=1}^{\infty }d_{i}(\frac{1}{i+2}-\frac{1}{2}) \end{array}$$

It can be seen from the Equation (41) that, in this pressurized blister test, the energy release rate *G* can be determined by measuring the radius *a* of the final blistering thin film and the height $h_{1,i}$ (*i* = 1, 2, 3, …, *n*) of colored liquid in the left circular container after each step of loading operation.

## 4. Results and Discussions

### 4.1. Improvements on Experimental Setup and Theoretical Derivation of Energy Release Rate

Compared with the experimental setup before improved, the two circular containers in the improved experimental setup have the same inner radius, and a valve is added to the connecting pipe to eliminate the influence of liquid fluctuation caused by pouring liquid into the right container. As it is known, the pressure *q* in the compressed air is independent of the inner radii of the two circular containers, but the inner radii of the two circular containers have a direct effect on the relationship between the heights of colored liquid in these two containers [22]. Therefore, if these two containers are designed with the same inner radius, it will not only have no effect on the pressure *q* in the compressed air, but also be helpful for simplifying following derivation process of the energy release rate, because the rising height of the colored liquid in the left container is exactly equal to the descending height of the colored liquid in the right container, as shown in Equation (2).

In the theoretical derivation of the energy release rate, some improvements are made and some errors in previous work are also revised, the main improvements and revisions are as follows:

1. The experimental setup was improved to make it more reasonable.
2. The work *U_F_*(*a*) is obtained by summing up the work *U_F,i_* caused by the change of liquid potential energy in each step of loading operation.
3. The compression of the enclosed air in the left circular container can be regarded as isothermal compression. So, the energy *U_a_*(*a*) absorbed when the enclosed air is compressed will be released into the atmosphere in the form of heat energy, instead of being stored in the enclosed air. From this point of view, the energy *U_a_*(*a*) can be obtained by calculating the energy released when the enclosed air is isothermal compressed.
4. The formula used to calculate the volume *V* under the blistering thin film is revised.
5. More importantly, a more accurate formula for determining the strain energy *U_ef_*(*a*) stored in the blistering film is derived out based on the obtained refined closed-form solution of the problem of axisymmetric deformation of the pressurized blistering thin film. Compared with the existing solutions of the well-known Hencky solution, the assumptions or approximations used in the out-of-plane equilibrium equation, in-plane equilibrium equation and geometric equation were simultaneously given up during the derivation of the presented refined closed-form solution.
6. Based on the above-mentioned improvements and revisions, a new and more refined theory for characterizing the adhesion between elastic coatings and rigid substrates is finally developed.

### 4.2. Experimental Verification and Discussion on the Refined Closed-Form Solution

The validity of the refined closed-form solution presented in this paper is of great important to determine the elastic strain energy *U_ef_*(*a*) stored in the blistering thin film. Next, an experiment was conducted to demonstrate the validity of the presented refined closed-form solution. In addition, the influence of the above-mentioned assumptions (i)–(iii) on the analytic solution of the well-known Hencky problem was also discussed.

**Figure 7:**
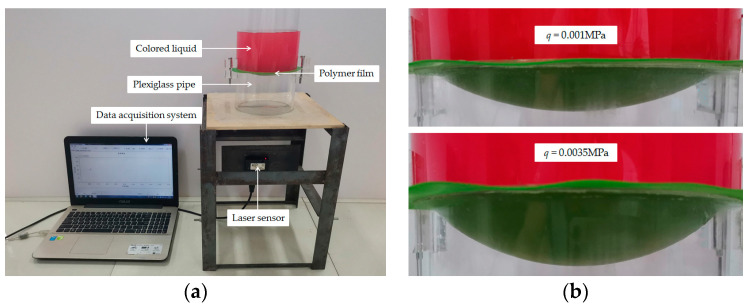
Photographs of an experiment on the large deflection deformation of a polymer thin film: (**a**) Experimental setup; (**b**) The deflected polymer thin film under different loads.

A polymer thin film with thickness h=0.7 mm, Young’s modulus of elasticity E=1.4 MPa, Poisson’s ratio ν=0.4 was clamped by two plexiglass pipes with inner radius 70 mm and wall thickness 5 mm, as shown in Figure 7a, where the non-contact laser displacement sensor was used to measure the deflection of the deflected thin film. After adding colored liquid (tap water mixed with a little red ink, the weight mix ratio is about 1000:1) into the upper plexiglass pipe, the polymer thin film was deformed and laterally deflected. The deflected polymer thin film, under different amounts of the colored liquid, is shown in Figure 7b, where *q* = 0.001 MPa and *q* = 0.0035 MPa denote the average loads applied onto the surface of the thin film (calculated by 1.570 kg and 5.495 kg, which are the weights of the added colored liquid), and the measured maximum deflections of the thin film are 18.66 mm (corresponding to 1.570 kg) and 29.17 mm (corresponding to 5.495 kg). The colored liquid of 1.570 kg is a cylinder with height 102 mm and radius 70 mm, and the colored liquid of and 5.495 kg is a cylinder with height 357 mm and radius 70 mm.

**Figure 8:**
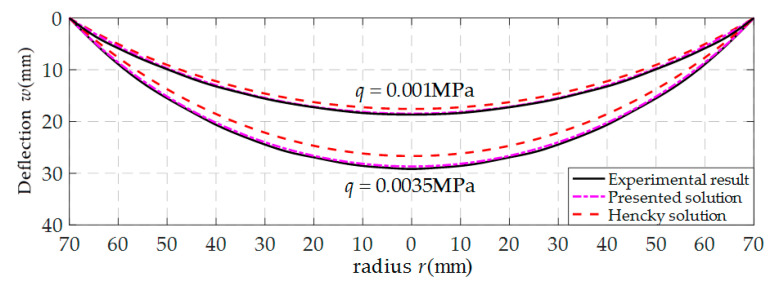
Variation of *w* with *r*.

Figure 8 shows the variations of the deflection *w* of the thin film with *r* when *q* takes 0.001 MPa and 0.0035 MPa respectively, where the solid lines represent the experimental results, the dash-dotted lines represent the results calculated by the refined closed-form solution presented here, and the dashed lines by the well-known Hencky solution (i.e., the solution used in Sun et al. [9]). From Figure 8 it can be seen that, the difference between the experimental results and the results calculated by the well-known Hencky solution is greater than that between the experimental results and the results calculated by the refined closed-form solution presented here, especially when *q* = 0.0035 MPa. This indicates that the calculation accuracy of the solution presented here is effectively improved due to the abandonment of the above-mentioned assumptions (i)–(iii) which are adopted during the derivation of the well-known Hencky solution. However, such a conclusion, it should also be noted, is obtained based on the experimental results of the experimental setup shown in Figure 7. The loading mode of the thin film in Figure 7 differs obviously from that in Figure 3 and Figure 4. The compressed air is applied onto the surface of thin film in Figure 3 and Figure 4, resulting in the always uniformly distributed loads acting on the surface of the thin film. Meanwhile, in Figure 7, the colored liquid acts directly on the surface of the thin film, resulting in no uniformly distributed loads acting on the surface of the thin film (see Figure 7b). For example, for *q* = 0.0035 MPa the actual height of the added colored liquid is measured to be 369.17 mm (the edge height 340 mm, the maximum deflection 29.17 mm) at the center of the circular thin film. While the loads used for the solutions, *q* = 0.0035 MPa, corresponds to the colored liquid of 5.495 kg, and corresponds to a cylinder with height 357 mm and radius 70 mm. The resulting loads error at the center of the circular thin film is about (369.17–357)/357 = 3.41%. Since this error is small, it does not cause qualitative change. Therefore, based on the results of the simple experiment conducted, it may be concluded that the refined closed-form solution presented in this paper is basically reliable.

**Figure 9:**
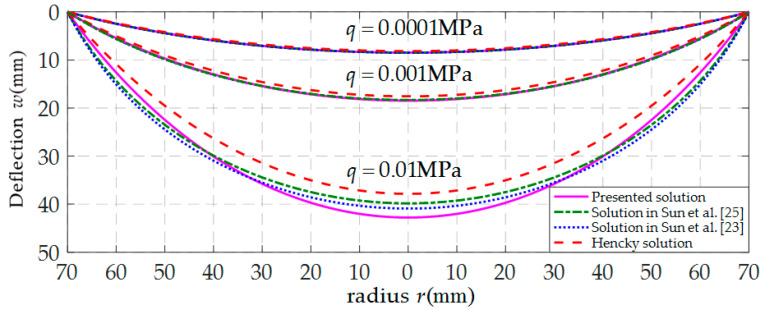
Variation of *w* with *r*.

In order to investigate the influence of the above-mentioned assumptions (i)–(iii) on the analytic solution of the well-known Hencky problem, the calculation results of the deflection of the polymer thin film obtained by using four existing analytical solutions of the well-known Hencky problem are presented, as shown in Figure 9, where the dashed lines represent the results obtained by the well-known Hencky solution (i.e., the solution used in Sun et al. [9], in which assumptions (i)–(iii) were all adopted), the dotted lines by the solution presented in Sun et al. [23] (only assumption (i) was given up), the dash-dotted lines by the solution presented in Sun et al. [25] (assumptions (i) and (ii) were simultaneously given up), and the solid lines by the refined closed-form solution presented here (assumptions (i)–(iii) were all given up). From Figure 9, it can be seen that the dashed line, dotted line, and the dash-dotted line are very close to the solid line when *q* takes 0.0001 MPa, which also demonstrates the validity of the refined closed-form solution presented here. Moreover, when *q* takes 0.001 MPa, the dotted line and dash-dotted line are still very close to the solid line, but the difference between the dashed line and solid line emerges; and this difference becomes more and more obvious when *q* takes 0.01 MPa. It can also be seen from Figure 9 that, compared with the dashed line, the dotted line and dash-dotted line is closer to the solid line when *q* takes 0.01 MPa, which means that the accuracy of the analytical solution of the well-known Hencky problem can be improve by giving up assumptions (i) and (ii); but compared with the dotted line, the accuracy of the dash-dotted line is reduced. Therefore, in order to obtain a more refined analytical solution, the assumptions (i)–(iii) all need to be given up, such that the elastic strain energy *U_ef_*(*a*) stored in the film can be calculated accurately.

**Figure 10:**
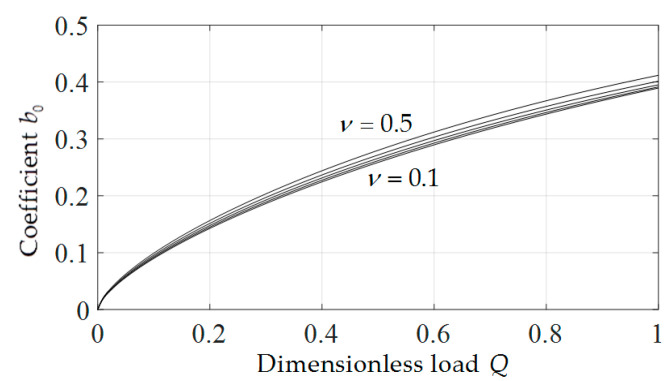
Variation of *b*_0_ with *Q*.

**Figure 11:**
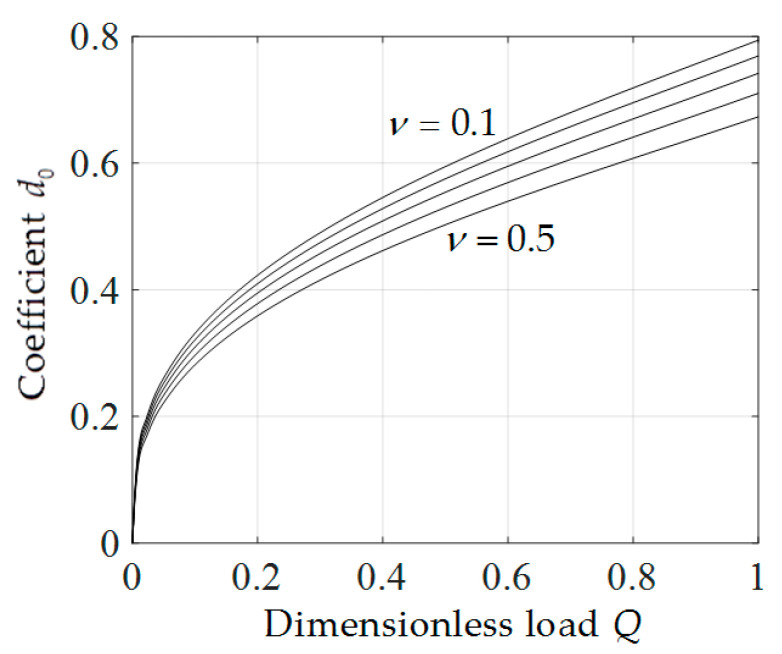
Variation of *d*_0_ with *Q*.

In addition, from Equations (32) and (34) it is found that, the coefficients $b_{0}$ and $d_{0}$ have important influence on the deflection and radial stress of the membrane at x=0, respectively. So, $b_{0}$ and $d_{0}$ are two very important parameters which are determined by the boundary conditions. When determining the undetermined constants $b_{0}$ and $d_{0}$, it is found that the values of $b_{0}$ and $d_{0}$ are only related to ν and *Q*. So, for the convenience of application, we presented the variations of $b_{0}$ and $d_{0}$ with *Q* when ν takes different values, as shown in Figure 10 and Figure 11. It can be seen from Figure 10 and Figure 11 that, $b_{0}$ and $d_{0}$ increase with the increase of *Q*, and under the same *Q*, $d_{0}$ decreases with the increase of *v* while $b_{0}$ increases with the increase of ν. In addition, it can also be seen that, compared with $b_{0}$, $d_{0}$ is more sensitive to ν, i.e., the influence of Poisson’s ratio ν on the deflection of membrane is greater than that on the radial stress of membrane.

### 4.3. The error of U_ef_(a) Brought by the Assumptions Used in the Well-Known Hencky Solution

In Sun et al. [9], the well-known Hencky solution is used to determine the elastic strain energy *U_ef_*(*a*) stored in the blistering film. However, the assumptions (i)–(iii) adopted in the well-known Hencky solution will inevitably bring errors to the calculation results of the elastic strain energy *U_ef_*(*a*). During the derivation of the refined closed-form solution presented here, the assumptions (i)–(iii) were all given up. So, the refined closed-form solution presented here should be more accurate than the well-known Hencky solution, and the result of the elastic strain energy *U_ef_*(*a*)calculated by the refined closed-form solution presented here should also be more accurate than that calculated by the well-known Hencky solution.

Suppose that a thin rubber film with thickness 0.06 mm, Young’s modulus of elasticity 7.84 MPa, Poisson’s ratio 0.47 is adhered to a rigid substrate, and a blistering thin film with radius of 20 mm appears when a loads *q* is applied on the thin film. When *q* takes different values, the coefficients of the expression of *W* (i.e., ***d_i_*** (*i* = 2,4,6,…)) in the well-known Hencky solution and in the refined closed-form solution presented here are listed Table 1. Then the elastic strain energy *U_ef_*(*a*) can be obtained by using Equation (40). From Table 1 it can be seen that, the elastic strain energy *U_ef_*(*a*) calculated by the well-known Hencky solution is smaller than that by the refined closed-form solution presented here, which will make the energy release rate *G* of delamination interfaces larger. It can also be found from Table 1 that, the difference between the calculated results of the elastic strain energy *U_ef_*(*a*) increase with the increase of the applied loads *q*. The errors of the *U_ef_*(*a*) brought by the assumptions adopted in the well-known Hencky solution are −1.5%, −6.4%, and −18.2% when *q* takes 0.001, 0.01, and 0.03 MPa, respectively. So, when the applied loads *q* is relatively large, the error of the *U_ef_*(*a*) brought by the assumptions or approximations adopted in the well-known Hencky solution cannot be ignored, and the formula of the *U_ef_*(*a*) obtained based on the well-known Hencky solution will gradually lose its effectiveness with the increase of the applied loads.

## 5. Concluding Remarks

The work reported here concerns the improvement in adhesion characterization theory based on the proposed pressurized blister test technique. The following are some conclusions made through this study.

The experimental setup for pressurized blister test has appropriately been improved, which results in the advantage of rationality and convenience in measurement and loading operation, in comparison with that in the previous work.

The static problem of mechanical behaviour of the blistering thin film is still simplified as the well-Known Hencky problem, as the previous work did, but the well-Known Hencky problem has here been reformulated, giving up some assumptions or approximations adopted in the derivation of the existing solutions (especially the well-Known Hencky solution), resulting in a new and more refined closed-form solution in comparison with the all existing solutions.

The new and more refined closed-form solution is, by way of the conducted experiment, proven to be reliable, and thus the developed, new, and more refined theory for characterizing adhesion between elastic coatings and rigid substrates, which is derived out by this new and more refined closed-form solution, should also be reliable to some extent.

## Figures and Tables

**Table 1:** The coefficients ***d_i_*** and the elastic strain energy *U_ef_*(*a*) stored in blistering film.

Analytic Solution	*q*(MPa)	*d_i_* (*i* = 2, 4, 6, …)						*U_ef_*(*a*) (×10^−3^ J)
		*d*_2_	*d*_4_	*d_6_*	*d*_8_	*d_10_*	*d_12_*	
Hencky solution	0.001	−0.1915	−0.0158	−0.0029	−0.0007	−0.0002	−0.00004	0.6862
	0.01	−0.4126	−0.0341	−0.0063	−0.0014	−0.0004	−0.0001	14.7842
	0.03	−0.5951	−0.0492	−0.0090	−0.0020	−0.0005	−0.0001	63.9382
Refined closed-form solution	0.001	−0.1913	−0.0162	−0.0039	−0.0012	−0.0005	−0.0002	0.6970
	0.01	−0.4237	−0.0435	−0.0074	−0.0031	−0.0019	−0.0011	15.7963
	0.03	−0.6927	−0.1166	0.0565	0.0550	−0.0302	−0.0605	78.1752

**Figure A1:**
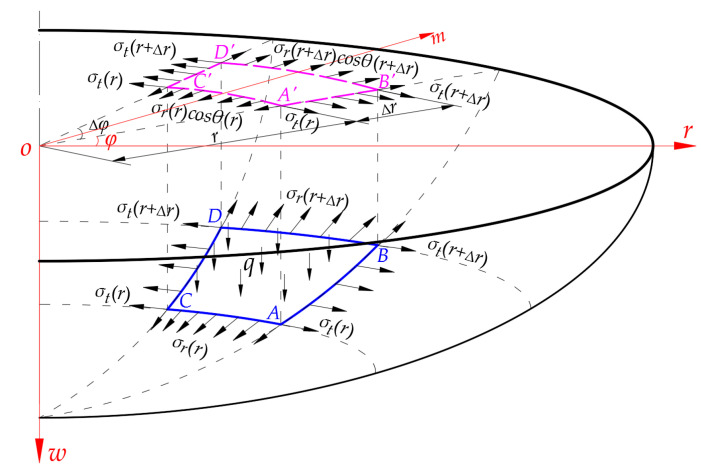
Sketch of the cylindrical coordinate system and the micro element *ABCD*.

**Figure A2:**
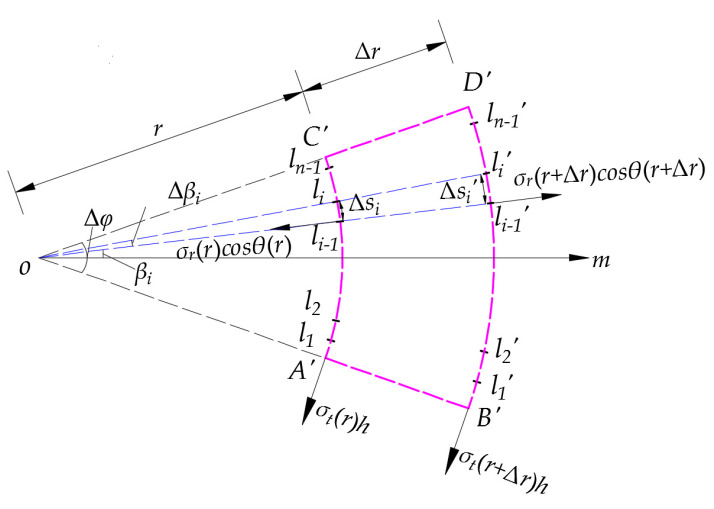
Diagram of the projection of the micro element and its stress.

## Nomenclature

*U_F_*	external work caused by the change of the potential energy of the liquid
*U_F_*(*a*)	the *U_F_* when *r* = *a*
*U_a_*	energy absorbed by the enclosed air
*U_a_*(*a*)	the U_a_ when *r* = *a*
*U_ef_*	elastic strain energy stored in the blistering thin film
*U_ef_*(*a*)	the *U_ef_* when *r* = *a*
*U_d_*	energy released on the delamination region
*G*	energy release rate
*K*	proportional coefficient
*S*	area of the circular delamination region
*a*	radius of the circular delamination region
*R*_0_	radius of the hole in the rigid substrate
*R*	inner radius of the container
*H*_1_	initial height of the air in left container
*H*	height of the liquid added to the right container each time
*h*_1,*i*_	height of liquid in the left container after step *i* of loading operation
*h*_2,*i*_	height of liquid in the right container after step *i* of loading operation
*ρ*	density of the liquid in the container
*g*	acceleration of gravity
*P*	pressure of gas
*V*	volume of gas
*N*	number of gas molecules per unit volume
*K*	Boltzmann constant
*T*	temperature of gas
*V*_0_	initial volume of the air in left container
*V^n^*	volume of the air in left container after the step *n* of loading operation
*r*	radial coordinate
*w*	transversal displacement
*u*	radial displacement
*h*	thickness of the membrane
*θ*	slope angle of the deflected membrane
*σr*	radial stress
*σt*	circumferential stress
*er*	radial strain
*et*	circumferential strain
*E*	Young’s modulus of elasticity
ν	Poisson’s ratio
*x*	dimensionless form of *r*
*Q*	dimensionless form of *q*
*W*	dimensionless form of *w*
*U*	dimensionless form of *u*
*S_r_*	dimensionless form of *σ_r_*
*S_t_*	dimensionless form of *σ_t_*
*b_i_*, *c_i_*, *d_i_*	coefficients of *Sr*, *St* and *W*

## Appendix A. Derivation of the In-Plane Equilibrium Equation

Taking the geometric middle plane of the undeformed circular membrane as the reference and taking the center point of the geometric middle plane as the coordinate origin, then the cylindrical coordinate system O-rφw is established. A micro element *ABCD* is taken from the deformed membrane to study the static equilibrium problem parallel to the radial coordinate direction, as shown in Figure A1, where *A′B′C′D′* is the projection of *ABCD* on the polar coordinates plane, $\sigma_{r}$ is the radial stress, $\sigma_{t}$ is the circumferential stress, θ is the angle between the radial stress and the polar coordinate plane (i.e., the slope angle of the deflected membrane).

In the radial coordinate direction, there are four radial forces on the micro element *ABCD*, i.e., the total forces *F*_1_ and *F*_2_ in the radial direction produced by the radial component of $\sigma_{r}$ acting on the edge $\overset{⌢}{AC}$ and $\overset{⌢}{BD}$ of micro element, and the total forces *F*_3_ and *F*_4_ in the radial direction produced by the radial component of $\sigma_{t}$ acting on the edge $\overset{⌢}{AB}$ and $\overset{⌢}{CD}$ of micro element, respectively. Moreover, the equilibrium equation of the micro element *ABCD* in the radial direction can be written as

(A1) $$F_{1}-F_{2}+F_{3}+F_{4}=0.$$

*F*_1_ and *F*_2_ can be determined from the *A′B′C′D′*, as shown in Figure A2, $\overset{⌢}{A^{\prime }C^{\prime }}$ is divided into *n* small arcs by points *l*_1_, *l*_2_, *l*_3_, …, *l_n−_*_1_, where $\bar{om}$ is the angular bisector of $∠A^{\prime }oC^{\prime }$. Supposing $\overset{⌢}{l_{i}l_{i-1}}$ is one of those small arcs, $\beta_{i}$ is the angle between $\bar{om}$ and the radial stress on the small arc $\overset{⌢}{l_{i}l_{i-1}}$, $\Delta s_{i}$ is the length of the small arc $\overset{⌢}{l_{i}l_{i-1}}$, then *F*_1_ can be written as

(A2) $$F_{1}\approx \sum_{i=1}^{n}\sigma_{r}(r)h\Delta s_{i}\cos\theta (r)\cos\beta_{i}=\sum_{i=1}^{n}\sigma_{r}(r)h\Delta \beta_{i}r\cos\theta (r)\cos\beta_{i}.$$

Supposing μ is the maximum value of $\Delta \beta_{i}$, that is

(A3) $$\mu =\max\{\Delta \beta_{1},\Delta \beta_{2},\Delta \beta_{3},\ldots ,\Delta \beta_{n-1}\}.$$

When μ tends to zero, taking the limit of Equation (A2) yields

(A4) $$F_{1}=\underset{\mu \to 0}{\lim}\sum_{i=1}^{n}\sigma_{r}(r)h\Delta \beta_{i}r\cos\theta (r)\cos\beta_{i}.$$

It is obvious that Equation (A4) is the definite integral of function $\sigma_{r}(r)hr\cos\theta (r)\cos\beta$ of variable β on interval [−Δφ/2,Δφ/2], so Equation (A4) can be written as

(A5) $$F_{1}\;=\sigma_{r}(r)hr\cos\theta (r)\int_{-\Delta \varphi /2}^{\Delta \varphi /2}\cos\beta d\beta \;=2\sigma_{r}(r)hr\cos\theta (r)\sin\frac{\Delta \varphi }{2}.$$

With the same approach, *F*_2_ can be written as

(A6) $$F_{2}=2\sigma_{r}(r+\Delta r)h(r+\Delta r)\cos\theta (r+\Delta r)\sin\frac{\Delta \varphi }{2}.$$

Since the micro element is infinitesimal, we can assume that the length of curve $\overset{⌢}{AB}$ is approximately equal to the length of straight line $\bar{AB}$, and $\sigma_{t}(r)$ changes linearly from $\sigma_{t}(r)$ to $\sigma_{t}(r+\Delta r)$ on edge $\overset{⌢}{AB}$ and $\overset{⌢}{CD}$. Therefore, *F*_3_ and *F*_4_ can be written as

(A7) $$F_{3}=F_{4}=\frac{\sigma_{t}(r)+\sigma_{t}(r+\Delta r)}{2}h\sqrt{{\left[w(r)-w(r+\Delta r)\right]}^{2}+{\left(\Delta r\right)}^{2}}\sin\frac{\Delta \varphi }{2}.$$

Substituting Equations (A5)–(A7) into Equation (A1), it is found that

(A8) $$\begin{array}{l} \sigma_{r}(r+\Delta r)h(r+\Delta r)\cos\theta (r+\Delta r)-\sigma_{r}(r)hr\cos\theta (r)\\ -\frac{\sigma_{t}(r)+\sigma_{t}(r+\Delta r)}{2}h\sqrt{{\left[w(r)-w(r+\Delta r)\right]}^{2}+{\left(\Delta r\right)}^{2}}=0 \end{array}$$

It is assumed that θ(r)≅θ(r+Δr), then Equation (A8) can be written as

(A9) $$\begin{array}{l} \sigma_{r}(r+\Delta r)h(r+\Delta r)\cos\theta (r)-\sigma_{r}(r)hr\cos\theta (r)\\ -\frac{\sigma_{t}(r)+\sigma_{t}(r+\Delta r)}{2}h\sqrt{{\left[w(r)-w(r+\Delta r)\right]}^{2}+{\left(\Delta r\right)}^{2}}=0 \end{array}$$

Substituting $\cos\theta =1/\sqrt{1+\tan\theta^{2}}=1/\sqrt{1+{\left(-w^{\prime }\right)}^{2}}$ into Equation (A9), it yields

(A10) $$\begin{array}{l} \sigma_{r}(r+\Delta r)h(r+\Delta r)\frac{1}{\sqrt{1+{\left[-w^{\prime }(r)\right]}^{2}}}-\sigma_{r}(r)hr\frac{1}{\sqrt{1+{\left[-w^{\prime }(r)\right]}^{2}}}\\ -\frac{\sigma_{t}(r)+\sigma_{t}(r+\Delta r)}{2}h\sqrt{{\left[w(r)-w(r+\Delta r)\right]}^{2}+{\left(\Delta r\right)}^{2}}=0 \end{array}$$

Expand $\sigma_{r}(r+\Delta r)$, $\sigma_{t}(r+\Delta r)$ and w(r+Δr) to the power series of the *r*, and neglect the terms that contain the second and higher orders of Δr

(A11) $$\left\{ \begin{array}{l} \sigma_{r}(r+\Delta r)=\sigma_{r}(r)+{\sigma^{\prime }}_{r}(r)\Delta r\\ \sigma_{t}(r+\Delta r)=\sigma_{t}(r)+{\sigma^{\prime }}_{t}(r)\Delta r\\ w(r+\Delta r)=w(r)+w^{\prime }(r)\Delta r \end{array}\right..$$

Substituting Equation (A11) into Equation (A10) and neglecting the terms that contain the second orders of Δr yields

(A12) $$\frac{\left[{\sigma^{\prime }}_{r}(r)\cdot r+\sigma_{r}(r)\right]}{\sqrt{1+{\left[-w^{\prime }(r)\right]}^{2}}}-\frac{\left[2\sigma_{t}(r)+{\sigma^{\prime }}_{t}(r)\Delta r\right]}{2}\sqrt{{\left[-w^{\prime }(r)-\frac{1}{2!}w^{\prime \prime }(r)\Delta r\right]}^{2}+1}=0.$$

When Δr→0, it is found that

(A13) $$[{\sigma^{\prime }}_{r}(r)\cdot r+\sigma_{r}(r)]-\sigma_{t}(r)\{1+{\left[-w^{\prime }(r)\right]}^{2}\}=0.$$

Equation (A13) can also be written as

(A14) $$\frac{d}{dr}(r\sigma_{r})-\sigma_{t}[1+{\left(\frac{dw}{dr}\right)}^{2}]=0.$$

Equation (A14) is the modified in-plane equilibrium equation established by taking in account the effect of the deflection of membrane, i.e., the Equation (15) used in this paper.

## Appendix B

Expressions of $b_{i}$:

$$b_{1}=b_{3}=b_{5}=b_{7}=b_{9}=\ldots =0$$

$$b_{2}=\frac{1}{64}\frac{Q^{2}[(2\nu^{2}+4\nu -6)b_{0}^{2}+(-2\nu -6)b_{0}+1]}{(\nu b_{0}-b_{0}-1)b_{0}^{2}}$$

$$\begin{array}{l} b_{4}=\frac{Q^{4}}{12288{\left(\nu b_{0}-b_{0}-1\right)}^{3}b_{0}^{5}}[(4\nu^{5}+20\nu^{4}-24\nu^{3}-88\nu^{2}+148\nu -60)b_{0}^{5}+(-12\nu^{4}-72\nu^{3}+264\nu \\ \;\;-180)b_{0}^{4}+(4\nu^{3}+108\nu^{2}+60\nu -172)b_{0}^{3}+(6\nu^{2}-64\nu -38)b_{0}^{2}+(-7\nu +21)b_{0}+2] \end{array}$$

$$\begin{array}{l} b_{6}=-\frac{Q^{6}}{4718592b_{0}^{8}{\left(\nu b_{0}-b_{0}-1\right)}^{5}}[(48\nu^{8}+336\nu^{7}-432\nu^{6}-2544\nu^{5}+4080\nu^{4}+3312\nu^{3}-10896\nu^{2}\\ \;\;+8812\nu -2016)b_{0}^{8}+(-240\nu^{7}-1920\nu^{6}+240\nu^{5}+12960\nu^{4}-7440\nu^{3}-24000\nu^{2}+30480\nu \\ \;\;-10080)b_{0}^{7}+(412\nu^{6}+5696\nu^{5}+396\nu^{4}-20704\nu^{3}-3404\nu^{2}+36384\nu -18780)b_{0}^{6}+(-440\nu^{5}\\ \;\;-9400\nu^{4}-432\nu^{3}+16016\nu^{2}+9064\nu -14808)b_{0}^{5}+(196\nu^{4}+10044\nu^{3}-396\nu^{2}-7084\nu -2760)b_{0}^{4}\\ \;\;+(64\nu^{3}-6508\nu^{2}+328\nu +1508)b_{0}^{3}+(-139\nu^{2}+2492\nu -365)b_{0}^{2}+(70\nu -414)b_{0}-13] \end{array}$$

$$\begin{array}{l} b_{8}=-\frac{Q^{8}}{3019898880b_{0}^{11}{\left(\nu b_{0}-b_{0}-1\right)}^{7}}[(3360\nu^{10}+24960\nu^{9}-80160\nu^{8}-199680\nu^{7}+840000\nu^{6}\\ \;\;-349440\nu^{5}-2103360\nu^{4}+4085760\nu^{3}-3354720\nu^{2}+1353600\nu -220320)b_{0}^{11}+(-23520\nu^{9}\\ \;\;-198240\nu^{8}+362880\nu^{7}+1760640\nu^{6}-4119360\nu^{5}-1673280\nu^{4}+13050240\nu^{3}-15550080\nu^{2}\\ \;\;+7932960\nu -1542240)b_{0}^{10}+(1144\nu^{9}+10392\nu^{8}+972096\nu^{7}-998912\nu^{6}-5469840\nu^{5}\\ \;\;+7437936\nu^{4}+10223488\nu^{3}-26362176\nu^{2}+18746712\nu -4560840)b_{0}^{9}+(3536\nu^{8}+159280\nu^{7}\\ \;\;-2551472\nu^{6}+1399344\nu^{5}+9325040\nu^{4}-6036976\nu^{3}-17004560\nu^{2}+21988752\nu -7282944)b_{0}^{8}\\ \;\;+(-11700\nu^{7}-575948\nu^{6}+4167164\nu^{5}-1060860\nu^{4}-9371740\nu^{3}+1693660\nu^{2}+11673108\nu \\ \;\;-6513684)b_{0}^{7}+(15080\nu^{6}+979400\nu^{5}-4425584\nu^{4}+382096\nu^{3}+5710216\nu^{2}+148136\nu \\ \;\;-2809344)b_{0}^{6}+(-7734\nu^{5}-1038294\nu^{4}+3202252\nu^{3}-52244\nu^{2}-2084822\nu -19158)b_{0}^{5}\\ \;\;+(-2064\nu^{4}+715572\nu^{3}-1522436\nu^{2}-9076\nu +357204)b_{0}^{4}+(5851\nu^{3}-319097\nu^{2}+451169\nu \\ \;\;-24635)b_{0}^{3}+(-3872\nu^{2}+83624\nu -61360)b_{0}^{2}+(1249\nu -9867)b_{0}-170] \end{array}$$

$$\begin{array}{l} b_{10}=\frac{Q^{10}}{2899102924800b_{0}^{14}{\left(\nu b_{0}-b_{0}-1\right)}^{9}}[(22400\nu^{14}+409920\nu^{13}+1014720\nu^{12}-9726080\nu^{11}\\ \;\;-3521280\nu^{10}+86385600\nu^{9}-111330240\nu^{8}-171037440\nu^{7}+582744960\nu^{6}-550034240\nu^{5}\\ \;\;+35112000\nu^{4}+348136320\nu^{3}-304353280\nu^{2}+112365120\nu -16188480)b_{0}^{14}+(-201600\nu^{13}\\ \;\;-3890880\nu^{12}-13023360\nu^{11}+74511360\nu^{10}+106202880\nu^{9}-671267520\nu^{8}+330704640\nu^{7}\\ \;\;+1870041600\nu^{6}-3374663040\nu^{5}+1575645120\nu^{4}+1259637120\nu^{3}-1873589760\nu^{2}\\ \;\;+865589760\nu -145696320)b_{0}^{13}+(877424\nu^{12}+13856448\nu^{11}+97175520\nu^{10}-341615296\nu^{9}\\ \;\;-489544432\nu^{8}+2072814464\nu^{7}+273949760\nu^{6}-6299616640\nu^{5}+6738156176\nu^{4}-14196288\nu^{3}\\ \;\;-4216050208\nu^{2}+2722935872\nu -558742800)b_{0}^{12}+(-1833472\nu^{11}-28624640\nu^{10}-377462272\nu^{9}\\ \;\;+970592000\nu^{8}+1233044480\nu^{7}-3745627648\nu^{6}-2152600576\nu^{5}+10025887232\nu^{4}\\ \;\;-5730388480\nu^{3}-3346656000\nu^{2}+4325224960\nu -1171555584)b_{0}^{11}+(2530256\nu^{10}+27659168\nu^{9}\\ \;\;+948859856\nu^{8}-1906270080\nu^{7}-2010922592\nu^{6}+4592590016\nu^{5}+3483742752\nu^{4}\\ \;\;-8686119296\nu^{3}+1747844240\nu^{2}+3209060512\nu -1408974832)b_{0}^{10}+(-2272896\nu^{9}+10991840\nu^{8}\\ \;\;-1652145088\nu^{7}+2664400960\nu^{6}+2358232384\nu^{5}-4169356544\nu^{4}-2792458560\nu^{3}\\ \;\;+4314276288\nu^{2}+146218560\nu -877886944)b_{0}^{9}+(1293024\nu^{8}-83384976\nu^{7}+2090074736\nu^{6}\\ \;\;-2716072144\nu^{5}-2029579664\nu^{4}+2826419792\nu^{3}+1222698832\nu^{2}-1193075440\nu \\ \;\;-118374160)b_{0}^{8}+(-412456\nu^{7}+144241880\nu^{6}-1941687272\nu^{5}+2014558744\nu^{4}+1251889736\nu^{3}\\ \;\;-1368033976\nu^{2}-231963960\nu +131407304)b_{0}^{7}+(223816\nu^{6}-151574252\nu^{5}+1326402684\nu^{4}\\ \;\;-1088660824\nu^{3}-512170784\nu^{2}+437971268\nu -12191908)b_{0}^{6}+(-430984\nu^{5}+108223300\nu^{4}\\ \;\;-651169928\nu^{3}+410820848\nu^{2}+124004176\nu -68861812)b_{0}^{5}+(514053\nu^{4}-53453864\nu^{3}\\ \;\;+219557418\nu^{2}-101677512\nu -8835455)b_{0}^{4}+(-350854\nu^{3}+17631250\nu^{2}-45524858\nu \\ \;\;+12106846)b_{0}^{3}+(145077\nu^{2}-3525540\nu +4378799)b_{0}^{2}+(-34588\nu +326224)+3700] \end{array}$$

Expressions of $c_{i}$:

$$c_{1}=c_{3}=c_{5}=c_{7}=c_{9}=\ldots =0$$

$$c_{0}=b_{0}$$

$$c_{2}=\frac{1}{64}\frac{Q^{2}[(6\nu^{2}-4\nu -2)b_{0}^{2}+(-6\nu -2)b_{0}+3]}{b_{0}^{2}(\nu b_{0}-b_{0}-1)}$$

$$\begin{array}{l} c_{4}=\frac{Q^{4}}{12288b_{0}^{5}{\left(\nu b_{0}-b_{0}-1\right)}^{3}}[(20\nu^{5}+4\nu^{4}-120\nu^{3}+136\nu^{2}-28\nu -12)b_{0}^{5}+(-60\nu^{4}-72\nu^{3}+288\nu^{2}\\ \;\;-120\nu -36)b_{0}^{4}+(20\nu^{3}+204\nu^{2}-180\nu -44)b_{0}^{3}+(30\nu^{2}-128\nu +2)b_{0}^{2}+(-35\nu +57)b_{0}+10] \end{array}$$

$$\begin{array}{l} c_{6}=-\frac{Q^{6}}{4718592b_{0}^{8}{\left(\nu b_{0}-b_{0}-1\right)}^{5}}[(336\nu^{8}+48\nu^{7}-3024\nu^{6}+2928\nu^{5}+5520\nu^{4}-11376\nu^{3}+6672\nu^{2}\\ \;\;-816\nu -288)b_{0}^{8}+(-1680\nu^{7}-1920\nu^{6}+13200\nu^{5}-1440\nu^{4}-29040\nu^{3}+27840\nu^{2}-5520\nu \\ \;\;-1440)b_{0}^{7}+(2884\nu^{6}+11072\nu^{5}-28332\nu^{4}-8992\nu^{3}+42988\nu^{2}-18336\nu -1284)b_{0}^{6}\\ \;\;+(-3080\nu^{5}-21448\nu^{4}+32688\nu^{3}+15344\nu^{2}-27560\nu +4056)b_{0}^{5}+(1372\nu^{4}+27108\nu^{3}\\ \;\;-25812\nu^{2}-12148\nu +9480)b_{0}^{4}+(448\nu^{3}-19348\nu^{2}+9784\nu +4508)b_{0}^{3}+(-973\nu^{2}+8228\nu \\ \;\;-3131)b_{0}^{2}+(490\nu -1458)b_{0}-91] \end{array}$$

$$\begin{array}{l} c_{8}=-\frac{Q^{8}}{3019898880b_{0}^{11}{\left(\nu b_{0}-b_{0}-1\right)}^{7}}[(30240\nu^{10}-44160\nu^{9}-299040\nu^{8}+814080\nu^{7}+33600\nu^{6}\\ \;\;-2607360\nu^{5}+4186560\nu^{4}-3010560\nu^{3}+988320\nu^{2}-67200\nu -24480)b_{0}^{11}+(-211680\nu^{9}\\ \;\;+97440\nu^{8}+2190720\nu^{7}-3507840\nu^{6}-3743040\nu^{5}+14508480\nu^{4}-14797440\nu^{3}+6276480\nu^{2}\\ \;\;-641760\nu -171360)b_{0}^{10}+(10296\nu^{9}+435928\nu^{8}+1203264\nu^{7}-7780608\nu^{6}+5382640\nu^{5}\\ \;\;+15869424\nu^{4}-29083008\nu^{3}+17070016\nu^{2}-2601192\nu -506760)b_{0}^{9}+(31824\nu^{8}-320080\nu^{7}\\ \;\;-5196848\nu^{6}+14270896\nu^{5}-554640\nu^{4}-27132784\nu^{3}+25800560\nu^{2}-6171632\nu -727296)b_{0}^{8}\\ \;\;+(-105300\nu^{7}-1026732\nu^{6}+10986076\nu^{5}-15582940\nu^{4}-7180860\nu^{3}+22791740\nu^{2}-9742028\nu \\ \;\;-139956)b_{0}^{7}+(135720\nu^{6}+2833800\nu^{5}-13212656\nu^{4}+10242064\nu^{3}+8499144\nu^{2}-9857176\nu \\ \;\;+1359104)b_{0}^{6}+(-69606\nu^{5}-3657446\nu^{4}+10626668\nu^{3}-4514996\nu^{2}-4646598\nu +2261978)b_{0}^{5}\\ \;\;+(-18576\nu^{4}+2798548\nu^{3}-5445924\nu^{2}+1057516\nu +1147636)b_{0}^{4}+(52659\nu^{3}-1342273\nu^{2}\\ \;\;+1770921\nu -284115)b_{0}^{3}+(-34848\nu^{2}+368616\nu -257840)b_{0}^{2}+(11241\nu -44803)b_{0}-1530] \end{array}$$

$$\begin{array}{l} c_{10}=-\frac{Q^{10}}{2899102924800b_{0}^{14}{\left(\nu b_{0}-b_{0}-1\right)}^{9}}[(246400\nu^{14}+1821120\nu^{13}-8460480\nu^{12}-12906880\nu^{11}\\ \;\;+93515520\nu^{10}-81950400\nu^{9}-236792640\nu^{8}+611976960\nu^{7}-492589440\nu^{6}-49147840\nu^{5}\\ \;\;+376824000\nu^{4}-280452480\nu^{3}+85227520\nu^{2}-5839680\nu -1471680)b_{0}^{14}+(-2217600\nu^{13}\\ \;\;-18607680\nu^{12}+57536640\nu^{11}+173698560\nu^{10}-667941120\nu^{9}+69612480\nu^{8}+2200746240\nu^{7}\\ \;\;-3307046400\nu^{6}+1126258560\nu^{5}+1568589120\nu^{4}-1822826880\nu^{3}+701245440\nu^{2}-65802240\nu \\ \;\;-13245120)b_{0}^{13}+(9651664\nu^{12}+72896448\nu^{11}-71144160\nu^{10}-1119267776\nu^{9}+2334557488\nu^{8}\\ \;\;+1241451904\nu^{7}-7695302720\nu^{6}+6999898240\nu^{5}+694820656\nu^{4}-4653734208\nu^{3}\\ \;\;+2633550112\nu^{2}-412307648\nu -35070000)b_{0}^{12}+(-20168192\nu^{11}-188320000\nu^{10}-269349632\nu^{9}\\ \;\;+3675105280\nu^{8}-4655667200\nu^{7}-5325813248\nu^{6}+14630393344\nu^{5}-7433028608\nu^{4}\\ \;\;-4361576960\nu^{3}+5412468480\nu^{2}-1487153920\nu +23110656)b_{0}^{11}+(27832816\nu^{10}+263480608\nu^{9}\\ \;\;+1578408496\nu^{8}-7878679680\nu^{7}+5949022688\nu^{6}+10909049536\nu^{5}-17558841888\nu^{4}\\ \;\;+4055022464\nu^{3}+5381205040\nu^{2}-3057657568\nu +331157488)b_{0}^{10}+(-25001856\nu^{9}\\ \;\;-160093280\nu^{8}-3755607488\nu^{7}+11562032960\nu^{6}-4672981696\nu^{5}-14016774784\nu^{4}\\ \;\;+14067898560\nu^{3}-553288512\nu^{2}-3242293440\nu +796109536)b_{0}^{9}+(14223264\nu^{8}-177285456\nu^{7}\\ \;\;+5772680176\nu^{6}-12292371344\nu^{5}+1847905136\nu^{4}+11989787152\nu^{3}-7398538288\nu^{2}\\ \;\;-610917680\nu +854517040)b_{0}^{8}+(-4537016\nu^{7}+541371400\nu^{6}-6112123192\nu^{5}+9363854984\nu^{4}\\ \;\;+258573976\nu^{3}-6649831976\nu^{2}+2412668760\nu +190023064)b_{0}^{7}+(2461976\nu^{6}-676309972\nu^{5}\\ \;\;+4625467524\nu^{4}-5212862504\nu^{3}-553817344\nu^{2}+2307859708\nu -492799388)b_{0}^{6}+(-4740824\nu^{5}\\ \;\;+527632460\nu^{4}-2460623128\nu^{3}+2014471888\nu^{2}+250199216\nu -404354012)b_{0}^{5}+(5654583\nu^{4}\\ \;\;-275673784\nu^{3}+889432878\nu^{2}-523295832\nu -3650965)b_{0}^{4}+(-3859394\nu^{3}+94353350\nu^{2}\\ \;\;-194684158\nu +65094986)b_{0}^{3}+(1595847\nu^{2}-19345020\nu +19427029)b_{0}^{2}\\ \;\;+(-380468\nu +1824464)b_{0}+40700] \end{array}$$

Expressions of $d_{i}$:

$$d_{1}=d_{3}=d_{5}=d_{7}=d_{9}=\ldots =0$$

$$d_{2}=-\frac{1}{4}\frac{Q}{b_{0}}$$

$$d_{4}=\frac{1}{512}\frac{Q^{3}[(2\nu^{2}-4\nu +2)b_{0}^{2}+(-2\nu +2)b_{0}+1]}{b_{0}^{4}(\nu b_{0}-b_{0}-1)}$$

$$\begin{array}{l} d_{6}=-\frac{Q^{5}}{147456b_{0}{}^{7}{\left(\nu b_{0}-b_{0}-1\right)}^{3}}[(8\nu^{5}-128\nu^{4}+240\nu^{3}-32\nu^{2}-184\nu +96)b_{0}^{5}+(-24\nu^{4}+360\nu^{3}-360\nu^{2}\\ \;\;-264\nu +288)b_{0}^{4}+(44\nu^{3}-420\nu^{2}+132\nu +244)b_{0}^{3}+(-42\nu^{2}+232\nu +2)b_{0}^{2}+(22\nu -60)b_{0}-5] \end{array}$$

$$\begin{array}{l} d_{8}=-\frac{Q^{7}}{75497472b_{0}^{10}{\left(\nu b_{0}-b_{0}-1\right)}^{5}}[(3216\nu^{7}-15408\nu^{6}+24912\nu^{5}-6000\nu^{4}-29520\nu^{3}+39024\nu^{2}\\ \;\;-20112\nu +3888)b_{0}^{8}+(-16080\nu^{6}+60960\nu^{5}-63600\nu^{4}-33600\nu^{3}+114000\nu^{2}-81120\nu \\ \;\;+19440)b_{0}^{7}+(-428\nu^{6}+38288\nu^{5}-108684\nu^{4}+60416\nu^{3}+94396\nu^{2}-124176\nu +40188)b_{0}^{6}\\ \;\;+(1336\nu^{5}-53608\nu^{4}+109296\nu^{3}-19600\nu^{2}-80936\nu +43512)b_{0}^{5}+(-2096\nu^{4}+47964\nu^{3}\\ \;\;-65748\nu^{2}-4012\nu +23892)b_{0}^{4}+(1948\nu^{3}-27136\nu^{2}+22492\nu +2696)b_{0}^{3}\\ \;\;+(-1117\nu^{2}+9128\nu -3815)b_{0}^{2}+(370\nu -1410)b_{0}-55] \end{array}$$

$$\begin{array}{l} d_{10}=\frac{Q^{9}}{60397977600b_{0}^{13}{\left(\nu b_{0}-b_{0}-1\right)}^{7}}[(1600\nu^{11}+72480\nu^{10}-960960\nu^{9}+3537120\nu^{8}-4771200\nu^{7}\\ \;\;-880320\nu^{6}+9475200\nu^{5}-9445440\nu^{4}+1308480\nu^{3}+3600800\nu^{2}-2431680\nu +493920)b_{0}^{11}\\ \;\;+(-11200\nu^{10}-518560\nu^{9}+6208160\nu^{8}-18551680\nu^{7}+14846720\nu^{6}+21008960\nu^{5}-45317440\nu^{4}\\ \;\;+20800640\nu^{3}+11641280\nu^{2}-13564320\nu^{2}+3457440)b_{0}^{10}+(23336\nu^{9}+1969928\nu^{8}-19393216\nu^{7}\\ \;\;+44830592\nu^{6}-13689520\nu^{5}-66084016\nu^{4}+71105792\nu^{3}-1117504\nu^{2}-27499192\nu \\ \;\;+9853800)b_{0}^{9}+(-17456\nu^{8}-4713840\nu^{7}+37036272\nu^{6}-63503984\nu^{5}-7305840\nu^{4}+89499696\nu^{3}\\ \;\;-45688880\nu^{2}-19459152\nu +14153184)b_{0}^{8}+(-33980\nu^{7}+7727068\nu^{6}-47283244\nu^{5}+57453900\nu^{4}\\ \;\;+25152780\nu^{3}-62541740\nu^{2}+10101212\nu +9424004)b_{0}^{7}+(116760\nu^{6}-8919720\nu^{5}+41681104\nu^{4}\\ \;\;-34006096\nu^{3}-21915336\nu^{2}+23022584\nu +20704)b_{0}^{6}+(-172946\nu^{5}+7335694\nu^{4}-25480732\nu^{3}\\ \;\;+12969284\nu^{2}+9123182\nu -3774482)b_{0}^{5}+(159544\nu^{4}-4242372\nu^{3}+10451396\nu^{2}-2963324\nu \\ \;\;-1562044)b_{0}^{4}+(-97581\nu^{3}+1657497\nu^{2}-2644289\nu +355355)b_{0}^{3}+(39292\nu^{2}-396104\nu \\ \;\;+31600)b_{0}^{2}+(-9469\nu +44047)b_{0}+1050] \end{array}$$
